# Optimization of the Expression Conditions of CGA-N46 in *Bacillus subtilis* DB1342(p-3N46) by Response Surface Methodology

**DOI:** 10.1007/s12539-015-0115-x

**Published:** 2015-09-04

**Authors:** Rui-Fang Li, Bin Wang, Shuai Liu, Shi-Hua Chen, Guang-Hai Yu, Shuo-Ye Yang, Liang Huang, Yan-Li Yin, Zhi-Fang Lu

**Affiliations:** College of Biological Engineering, Henan University of Technology, Zhengzhou, 450001 China

**Keywords:** *Bacillus subtilis* engineered strain, Genetic engineering expression, CGA-N46, Culture conditions optimization, Two-level factorial experiments, Response surface methodology, Biostatistic computer software, Biostatistic analysis, Antifungal peptide

## Abstract

CGA-N46 is a small antifungal-derived peptide and consists of the 31st–76th amino acids of the N-terminus of human chromogranin A. Polycistronic expression of recombinant CGA-N46 in *Bacillus subtilis* DB1342 was used to improve its production, but the yield of CGA-N46 was still low. In the present study, response surface methodology (RSM) was used to optimize culture medium composition and growth conditions of the engineered strain *B. subtilis* DB1342(p-3N46) for the further increase in CGA-N46 yield. The results of two-level factorial experiments indicated that dextrin and tryptone were significant factors affecting CGA-N46 expression. Central composite design (CCD) was used to determine the ideal conditions of each significant factors. From the results of CCD, the optimal medium composition was predicted to be dextrin 16.6 g/L, tryptone 19.2 g/L, KH_2_PO_4_·H_2_O 6 g/L, pH 6.5. And the optimal culture process indicated inoculation of *B. subtilis* DB1342(p-3N46) seed culture into fresh culture medium at 5 % (v/v), followed by expression of CGA-N46 for 56 hours at 30 °C induced by 2 % (v/v) sucrose after one hour of shaking culture. To test optimal CGA-N46 peptide expression, the yeast growth inhibition assay was employed and it was found that under optimal culture conditions, CGA-N46 inhibited the growth of *Candida albican* by 42.17, 30.86 % more than that in the pre-optimization conditions. In summary, RSM can be used to optimize expression conditions of CGA-N46 in engineered strains *B. subtilis* DB1342(p-3N46).

## Introduction

*Candida albican*, a ubiquitously distributed opportunistic pathogen, is the leading cause of candidas and causes one of the highest numbers of deaths among patients with fungal infections in the world [1, 2, 3]. Azole drugs are commonly used to treat infections caused by *C. albican*. However, some *Candida* spp. are intrinsically resistant or have reduced susceptibility to antifungal agents [4, 5, 6]. Antimicrobial peptides (AMPs) are multidimensionality powerful host shield and are hard for microorganisms to overcome using single approach resistance strategies. AMPs have shown to be effective alternatives to the current antimycotic therapies with the increasing resistance to conventional antimycotic drugs [7, 8]. CGA-N46, a peptide containing the 31st–76th amino acid of human chromogranin A, has antagonistic activity to *C. albican* [9]. To meet the need for drug development for future clinical applications, an abundance of CGA-N46 is necessary.

There are numerous approaches to optimizing recombinant expression. They are mainly two classes, i.e., genetic engineering and culture conditions optimization. The approaches on high efficient expression optimization mainly were genetic engineering, such as fusion expression [10, 11], induced expression [12, 13], or targeted codon optimization [14]. Culture conditions optimization were conducted in high-density cultivation [13, 15]. Li et al. [16] previously constructed an inducible tri-cistronic expression plasmid p-3N46 containing CGA-N46, which allowed three copies of *cga-*N46 gene be induced to express in one plasmid. Using this genetic strain, the expression of *cga-*N46 gene was increased by optimizing plasmid.

Production yield and cost of recombinant proteins are considerably influenced by bacterial culture conditions and medium composition. Optimization of culture conditions using biostatistic methods is another method to increase the expression of an engineered strain. Because of the difficulty of purification, one-factor-at-a-time was usually used [17]. However, this method frequently failed to locate the region of optimal response. Response surface methodology (RSM), exploring the relationships between several explanatory variables and one or more response variables [18], is characterized by more variables, fewer experiments, shorter cycle, and higher accuracy than other statistical optimization techniques such as one-factor-at-a-time [19, 17] or orthogonal tests. The main idea of RSM is to use a sequence of designed experiments to obtain an optimal response by using a second-degree polynomial model. As an optimization method, the experimental random error is considered in RSM, which is not in other traditional biostatistic methods. RSM is a simple, useful, and more precise methodology that can determine optimal culture medium composition and culture conditions using biostatistic computer software. RSM is commonly used for enhancing microorganism metabolite production at present [20, 21, 22, 18]. However, it is rarely reported in engineered strain expression.

In this study, RSM was used to optimize the medium and culture conditions for maximal expression of CGA-N46. To quantify CGA-N46, its effectiveness on inhibiting *C. albican* growth were tested.

## Methods

### Microorganisms

The engineered strains *B. subtilis* DB1342(pSBPTQ) which bore plasmid pSBPTQ [23] and *B. subtilis* DB1342(p-3N46) which bore plasmid p-3N46 [17] used in this study were previously constructed.

**Fig. 1:**
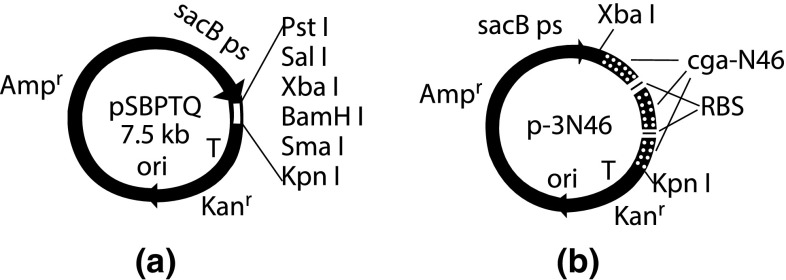
Physical map of pSBPTQ (**a**) and p-3N46 (**b**)

Plasmid pSBPTQ, an *Escherichia coli* and *Bacillus subtilis* shuttle plasmid, was a cloning plasmid with ampicillin resistance (*Amp* ^*r*^) in *E. coli* and an expression plasmid with kanamycin resistance (*Kan* ^*r*^) in *B. subtilis*. It had *sac*B promoter sequence (*sac*B *ps*) which can be induced by sucrose and promote the transcription of the exogenous gene downstream of *sac*B *ps*. It had multicloning sites between *sac*B *ps* and terminator sequence (*T*) which allowed inserting exogenous gene in pSBPTQ. The terminator could make an efficient stop of the transcription. Plasmid p-3N46 was a recombinant vector from pSBPTQ which bore three cistrons of *cga*-N46 gene between *sac*B *ps* and terminator in pSBPTQ. Each *cga*-N46 cistron had one ribosome binding site, one initiating codon, one copy of *cga*-N46 gene, one stop codon. Three cistrons of *cga*-N46 gene allowed three copies of CGA-N46 to be expressed in a single plasmid p-3N46. Their physical maps are shown in Fig. 1.

Yeast strain *C. albican* CSC314 was supplied by the Institute of Dermatology, Chinese Academy of Medical Sciences.

### Culture Media and Growth Conditions

Luria-Bertani (LB) agar medium (10 g tryptone, 5 g yeast extract, 10 g NaCl, 18 g agar in 1000 mL of distilled water, pH 7.0) containing 10 μg/mL kanamycin was used to culture *B. subtilis* DB1342 (pSBPTQ) and *B. subtilis* DB1342(p-3N46). The LB broth medium was prepared with the components as LB agar medium except agar as the growing culture for bacteria.

2 × MSR medium [24], contained glucose 10 g, tryptone 30 g, yeast extract 50 g, KH_2_PO_4_ 6 g in 1000 mL of distilled water, was used for expression culture optimization studies.

YPD medium (yeast extract 10 g, peptone 20 g, dextrose 20 g in 1000 mL of distilled water) was used to culture *C. albican* CSC314.

The seed culture was prepared by transferring a loopful of fresh transformed strains *B. subtilis* DB1342(pSBPTQ) and *B. subtilis* DB1342(p-3N46) cells into 25 ml LB broth in 250 mL Erlenmeyer flask. The flask was incubated at 250 rpm at 37 °C for 16 h.

### Expression Conditions

The production process was carried out, unless otherwise mentioned, in Erlenmeyer flasks (500 mL) containing 100 mL aliquots of the basal culture medium with 10 μg/mL kanamycin. After cultivated for 2 h at 37 °C 200 rpm, 5 mL 40 % sterile sucrose solution was added into culture medium to induce the expression of CGA-N46 for 48 h. During this period, CGA-N46 was expressed and secreted into culture supernatant. After expression, both of the culture were centrifuged at 12,000×*g* for 10 min at 4 °C. For antagonistic activity determination, 10 mL culture supernatant was filtered with 0.22 μm filter film.

### Assay of Antagonistic Activity

To analyze the antagonistic activity, 10 mL supernatant was mixed with equal volume of sterile YPD medium. *C. albican* (10^6^ cells/mL) was inoculated into the mixture medium at 10 % (v/v) and then incubated at 26 °C, 200 rpm. The supernatant from *B. subtilis* DB1342(pSBPTQ) was used as control. After incubating for 24 h, the yeast cells were centrifuged at 10,000×*g* for 10 min at room temperature. The cell precipitates were dried in a constant temperature oven at 37 °C to remove all traces of water. The dried cell precipitates were weighed, and yeast growth inhibition caused by CGA-N46 was calculated according to Eq. (1).

1 $$\begin{aligned} Y=(W_{2}-W_{1})/W_{2}\times 100\,\% \end{aligned}$$

Here, *W*_1_ was the yeast cell dry weight in experimental group, *W*_2_ was the yeast cell dry weight in control group, and *Y* was the indicator of yeast growth inhibition rate.

All experiments were conducted in triplicate, and the mean values were reported with standard error.

### Carbon Source Screening

The carbon source in $2\times \hbox {MSR}$ medium was replaced by glycerol, maltose, lactose, dextrin, and soluble starch, respectively, to optimize the carbon source of the culture medium. *B. subtilis* DB1342(p-3N46) seed culture was inoculated into the carbon source modified $2\times \hbox {MSR}$ medium to express CGA-N46. Yeast growth inhibition rates of *B. subtilis* DB1342(p-3N46) culture supernatant from each medium were measured.

### Nitrogen Source Screening

To study the effect of nitrogen source of the culture medium on the antagonistic activity of culture supernatant, the nitrogen source in $2\times \hbox {MSR}$ medium was replaced by casein, peptone, KNO_3_, ammonium sulfate, and urea, respectively. *B. subtilis* DB1342(p-3N46) seed culture was inoculated into nitrogen source modified $2\times \hbox {MSR}$ medium to express CGA-N46. Yeast growth inhibition rates of *B. subtilis* DB1342(p-3N46) culture supernatant from each medium were measured.

### Computer Software for Experimental Design and Statistical Analysis

The computer software, Design Expert 7.1 (Stat-Ease, Inc, Minneapolis, MN), was used for experimental design and regression analysis of the experimental data. For analysis of the fitted response nature and prediction of the maximum point, second-order equation was reduced to its canonical form.

### Response Surface Methodology for Optimizing Culture Conditions

#### Two-Level Factorial Experiment

Two-level factorial experiments were used to find significant factors. Considering the culture conditions reported in previous studies, the main factors in this study were set as follow (as shown in Table 1): dextrin (*A*), tryptone (*B*), initial medium pH (*C*), seed culture inoculation concentration (*D*), sucrose added time (*E*), induction period (*F*), and induction temperature (*G*). Design Expert 7.1 was used to design the experiments. Yeast growth inhibition rate (*Y*) was designed as response values. The significant factors affecting the expression of CGA-N46 would be determined according to the response values of different experimental conditions. Each experiment was conducted in three replicates.

**Table 1:** Two-level factorial experiment design and results

Run	*A* (g/L)	*B* (g/L)	*C*	*D* (%)	*E* (h)	*F* (h)	G (°C)	*Y* (%)
1	−1 (10)	−1 (10)	−1 (6.5)	−1 (5)	−1 (1)	−1 (48)	−1 (30)	42.24 ± 0.77
2	+1 (40)	−1 (10)	−1 (6.5)	−1 (5)	−1 (1)	+1 (56)	+1 (37)	18.31 ± 0.41
3	−1 (10)	+1 (40)	−1 (6.5)	−1 (5)	+1 (2)	+1 (56)	−1 (30)	16.8 ± 0.56
4	+1 (40)	+1 (40)	−1 (6.5)	−1 (5)	+1 (2)	−1 (48)	+1 (37)	−7.82 ± 2.04
5	−1 (10)	−1 (10)	−1 (6.5)	+1 (10)	+1 (2)	+1 (56)	+1 (37)	34.75 ± 1.12
6	+1 (40)	−1 (10)	−1 (6.5)	+1 (10)	+1 (2)	−1 (48)	−1 (30)	15.3 ± 0.38
7	−1 (10)	+1 (40)	−1 (6.5)	+1 (10)	−1 (1)	−1 (48)	+1 (37)	19.07 ± 0.25
8	+1 (40)	+1 (40)	−1 (6.5)	+1 (10)	−1 (1)	+1 (56)	−1 (30)	6.7 ± 0.44
9	−1 (10)	−1 (10)	+1 (7.5)	−1 (5)	+1 (2)	−1 (48)	+1 (37)	27.27 ± 0.73
10	+1 (40)	−1 (10)	+1 (7.5)	−1 (5)	+1 (2)	+1 (56)	−1 (30)	22.38 ± 0.40
11	−1 (10)	+1 (40)	+1 (7.5)	−1 (5)	−1 (1)	+1 (56)	+1 (37)	18.84 ± 0.21
12	+1 (40)	+1 (40)	+1 (7.5)	−1 (5)	−1 (1)	−1 (48)	−1 (30)	−0.98 ± 0.89
13	−1 (10)	−1 (10)	+1 (7.5)	+1 (10)	−1 (1)	+1 (56)	−1 (30)	31.26 ± 0.74
14	+1 (40)	−1 (10)	+1 (7.5)	+1 (10)	−1 (1)	−1 (48)	+1 (37)	13.08 ± 3.99
15	−1 (10)	+1 (40)	+1 (7.5)	+1 (10)	+1 (2)	−1 (48)	−1 (30)	15.98 ± 0.46
16	+1 (40)	+1 (40)	+1 (7.5)	+1 (10)	+1 (2)	+1 (56)	+1 (37)	−6.6 ± 3.83
17	0 (25)	0 (25)	0 (7.0)	0 (7.5)	0 (1.5)	0 (52)	0 (33)	17.42 ± 0.15
18	0 (25)	0 (25)	0 (7.0)	0 (7.5)	0 (1.5)	0 (52)	0 (33)	17.07 ± 0.74
19	0 (25)	0 (25)	0 (7.0)	0 (7.5)	0 (1.5)	0 (52)	0 (33)	23.37 ± 0.02
20	0 (25)	0 (25)	0 (7.0)	0 (7.5)	0 (1.5)	0 (52)	0 (33)	19.3 ± 0.26

*A*—dextrin, *B*—tryptone, *C*—medium pH, *D*—seed culture inoculation concentration, *E*—sucrose added time, *F*—induction period, *G*—induction temperature, *Y*—yeast growth inhibition rate

#### The Steepest Descent Experiment

In RSM, the optimal area of single significant factors could be further explored by designing the steepest descent experiments based on the results of two-level factorial experiments. It is also why RSM has higher accuracy than other statistical optimization techniques. These experimental designs are shown in Table 3. The optimal area of significant factors would be determined based on the response values.

#### Central Composite Design

In RSM, after the steepest ascent path experiments of single significant factors, the optimal level of significant factors would be further studied by central composite experiments. Central composite experiment design is shown in Table 4.

**Fig. 2:**
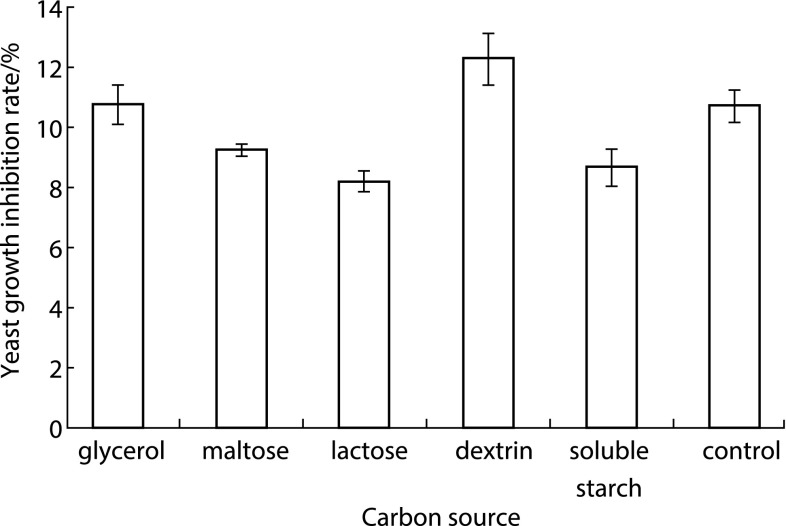
Effect of different carbon source in modified $2\times \hbox {MSR}$ medium on yeast growth inhibition rate of *B. subtilis* DB1342 (p-3N46) culture supernatant

## Results and Discussion

### Effect of Carbon Source on Yeast Growth Inhibition Rate

Yeast growth inhibition rates of *B. subtilis* DB1342(p-3N46) culture supernatant from each carbon source modified $2\times \hbox {MSR}$ media were calculated, and the results are presented in Fig. 2. The maximum yeast growth inhibition rate was 12.23 % in dextrin modified $2\times \hbox {MSR}$ medium, which increased 1.59 % than that of the control (10.64 %). Therefore, dextrin was the optimal carbon source for CGA-N46 expression.

### Effect of Nitrogen Source on Yeast Growth Inhibition Rate

**Fig. 3:**
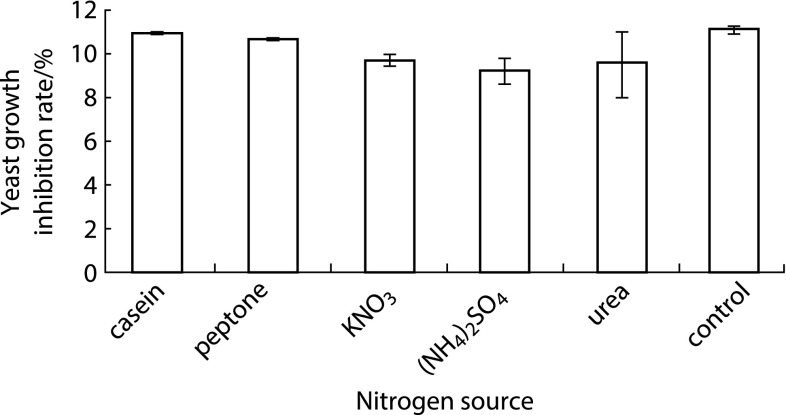
Effect of different nitrogen source in modified $2\times \hbox {MSR}$ medium on yeast growth inhibition rate of *B. subtilis* DB1342(p-3N46) culture supernatant

Yeast growth inhibition rates of *B. subtilis* DB1342(p-3N46) culture supernatant from each nitrogen source modified $2\times \hbox {MSR}$ media were calculated, and the results are shown in Fig. 3. The maximum yeast growth inhibition rate was 11.07 % with tryptone as nitrogen resource (the control). Tryptone was chosen to be the optimal nitrogen source for CGA-N46 expression.

### Yeast Growth Inhibition Rate in Pre-optimization Conditions

From the results of one-factor-at-a-time method for screening carbon and nitrogen sources, the culture medium composition was obtained as follows: yeast extract 50 g/L, dextrin 10 g/L, tryptone 30 g/L, KH_2_PO_4_·H_2_O 6 g/L. This culture medium was considered as pre-optimization medium. The antagonistic activity of *B. subtilis* DB1342(p-3N46) culture supernatant in pre-optimization conditions was calculated to be 11.31 %.

### Significant Factors

 There were 20 different experiments designed by software Design Expert 7.1 for two-level factorial experiment. The experiment results (i.e., response values) are shown in Table 1. The main effect analysis of the influencing factors is shown in Table 2. Dextrin and tryptone were two statistically significant factors (*p* < 0.05) that affected the yeast growth inhibition rate of *B. subtilis* DB1342(p-3N46) culture supernatant.

**Table 2:** Main effect analysis of two-level factorial experiment

Source	Sum of squares	*df*	Mean square	*F* value	*p* value (Prob > *F*)	Significance
*A*	1047.66	1	1047.66	24.89	0.0004	***
*B*	1426.76	1	1426.76	33.90	0.0001	***
*C*	17.04	1	17.04	0.40	0.5376	
*D*	11.04	1	11.04	0.261	0.6187	
*E*	23.60	1	23.60	0.56	0.4697	
*F*	25.38	1	25.38	0.60	0.4538	
*G*	162.37	1	162.37	3.86	0.0753	

*** indicate most significant values

The multiple regression equation of the response values (yeast growth inhibition rate) for the independent variables was obtained by software analysis.

2 $$\begin{aligned} Y&=13.94-8.09A-9.44B-1.03C-0.83D \nonumber \\&\quad-1.26E+1.21F-3.19G \end{aligned}$$

In Eq. (2), the independent variables included dextrin (*A*), tryptone (*B*), medium pH (*C*), seed culture inoculation concentration (*D*), sucrose added time (*E*), induction period (*F*), and induction temperature (*G*). From the coefficients of different variables in Eq. (2), the statistically nonsignificant factors, including medium pH, seed culture inoculation concentration, sucrose added time, and induction temperature were negative factors for response value, while the statistically nonsignificant factor induction period was positive one. Therefore, the −1 level for negative factors and the +1 level for positive factor were chosen for subsequent experiments. The values of the statistically nonsignificant factors were as follows: pH 6.5, the seed culture inoculation concentration 5 % (v/v), sucrose added after the seed culture inoculated for 1 h, induction temperature 30 °C, and induction period 56 h.

### The Steepest Descent Path Test Results of Significant Factors

The coefficients of significant factors dextrin (*A*) and tryptone (*B*) in Eq. (2) suggested that reducing the concentrations of dextrin and tryptone could improve the response value (yeast growth inhibition rate). The steepest descent path tests of single factor were designed to further optimize the significant factors. As initiation concentration of the descent path, 40 g/L dextrin and tryptone were chosen , and 5 g/L was set as the step length. The results in Table 3 showed that the response value of *B. subtilis* DB1342(p-3N46) supernatant in run 6 (dextrin 15 g/L and tryptone 15 g/L) was 41.11 %, suggesting that the optimal concentrations of dextrin and tryptone for response value were near 15 g/L.

**Table 3:** Steepest ascent experiment design and results

Run	*A* (g/L)	*B* (g/L)	Y (%)
1	40	40	9.06 ± 0.24
2	35	35	12.73 ± 2.00
3	30	30	9.73 ± 1.02
4	25	25	30.877 ± 1.88
5	20	20	36.90 ± 1.48
6	15	15	41.11 ± 1.87
7	10	10	34.76 ± 2.50
8	5	5	33.16 ± 2.29

*A*—dextrin, *B*—tryptone, *Y*—yeast growth inhibition rate

**Table 4:** Central composite experiment design and results

Run	*A* (g/L)	*B* (g/L)	*Y* (%)
1	−1 (10)	+1 (20)	34.78 ± 2.18
2	+1 (20)	−1 (10)	14.93 ± 1.20
3	−1 (10)	−1 (10)	25.12 ± 1.05
4	+1 (20)	+1 (20)	36.52 ± 0.98
5	−*α* (7.9)	0 (15)	26.3 ± 0.72
6	+*α* (22.1)	0 (15)	24.55 ± 1.50
7	0 (15)	−*α* (7.9)	17.61 ± 0.82
8	0 (15)	+*α* (22.1)	45.05 ± 1.18
9	0 (15)	0 (15)	41.03 ± 0.43
10	0 (15)	0 (15)	41.54 ± 0.45
11	0 (15)	0 (15)	41.76 ± 0.42
12	0 (15)	0 (15)	41.75 ± 0.38

*A*—Dextrin, *B*—Tryptone, *Y*—Yeast growth inhibition rate

### Central Composite Experimental Results

To get the exact optimal concentrations of significant factors, central composite experiments were performed. 15 g/L dextrin and 15 g/L tryptone were set as the central value. The levels of dextrin and tryptone and central composite experiment designs are shown in Table 4. A regression equation was obtained for response value when RSM analysis was used to evaluate dextrin and tryptone variables.

3 $$Y=41.13-0.63A+7.07B+9.24AB-7.63A^2-4.68B^2$$

**Fig. 4:**
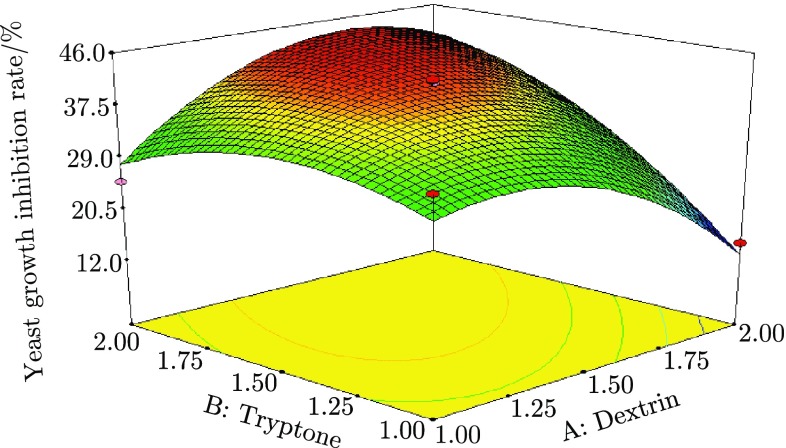
Three-dimensional surface curve showing the effect of dextrin and tryptone interaction on yeast growth inhibition rate

The variance of regression equation was analyzed by software Design Expert 7.10. The results are shown in Table 5 and Fig. 4.

**Table 5:** AVONA

Source	Sum of squares	*df*	Mean square	*F* value	*p* value (Prob > *F*)
Model	1183.82	5	1183.82	24.79	0.0006
*A*	3.22	1	3.22	0.34	0.5866
*B*	400.25	1	400.25	0.03	0.0006
*AB*	341.14	1	341.14	35.37	0.0010
*A* ^2^	372.89	1	372.89	0.99	0.0008
*B* ^2^	140.21	1	140.21	0.53	0.0086

The interaction effect of significant variables (dextrin and tryptone) on the response value (yeast growth inhibition rate) was studied by plotting three-dimensional surface curve. The three-dimensional curve of the calculated response (yeast growth inhibition rate) and contour plot from the interaction between the variables are shown in Fig. 4. The optimal concentration of dextrin and tryptone were 16.6 and 19.2 g/L, respectively. The theoretical maximum value of the yeast growth inhibition rate of DB1342(p-3N46) culture supernatant was 43.98 %.

### Validation of Experimental Model

*Bacillus subtilis* DB1342(p-3N46) and DB1342(pSBPTQ) were cultured under the optimized conditions. The yeast growth inhibition rate of *B. subtilis* DB1342(p-3N46) supernatant was 42.17 %, which was very close to the theoretical result 43.98 %. The result suggested that the experimental model was quite accurate. The results also showed that the inhibition rate of DB1342(p-3N46) supernatant from optimal conditions was significantly increased compared to the original conditions.

Compared with genetic engineering, the enhanced yield of CGA-N46 in culture conditions optimization was less, but it could be a valuable compensation to genetic engineering. Moreover, culture conditions optimization is a method to reduce the cost of expression for CGA-N46 industrial development.

This paper focused on investigation of significant factors that were responsible for peptide CGA-N46 expression in *B. subtilis* engineered strain DB1342(p-3N46) using mathematical and computational methods. The factors, such as nitrogen source and carbon source of medium components, initial medium pH, inoculum size, sucrose added time, and induction period, were considered. Although more factors were considered in this investigation than in other reports [25, 26], some other factors, such as metallic ions, could further improve engineered strain’s expression. But for the facilitation of the purification, the medium needs to be as simple as possible. Therefore, only some basal culture factors were chosen as the variables in the present investigation.

## Conclusion

Response surface methodology (RSM) experimental designs offered an efficient and feasible approach for culture conditions optimization of CGA-N46 expression in *B. subtilis* DB1342(p-3N46). The optimal medium composition was yeast 50 g/L, dextrin 16.6 g/L, tryptone 19.2 g/L, KH_2_PO_4_·H_2_O 6 g/L, pH6.5. The optimal culture conditions were inoculating seed culture at concentration of 5 % (v/v), adding sucrose up to 2 % one hour after the seed culture was inoculated, and allowing cultures to incubate at 30 °C for 56 h. Under optimal culture conditions, yeast growth inhibition rate of CGA-N46 was 31.53 % higher than that in original conditions. It was concluded that RSM was a method for efficiently improving the production of CGA-N46 in the engineered strain *B. subtilis* DB1342(p-3N46) and might provide an alternative approach to enhance the recombinant protein productivity in other engineered strains.
